# Empyema due to Streptococcus Pneumoniae Serotype 9V in a Child Immunized with 13-Valent Conjugated Pneumococcal Vaccine

**DOI:** 10.4274/balkanmedj.2015.0937

**Published:** 2017-01-05

**Authors:** Murat Sütçü, Hacer Aktürk, Fatih Karagözlü, Ayper Somer, Nezahat Gürler, Nuran Salman

**Affiliations:** 1 Department of Pediatric Infectious Diseases, İstanbul University İstanbul School of Medicine, İstanbul, Turkey; 2 Department of Clinical Microbiology, İstanbul University İstanbul School of Medicine, İstanbul, Turkey

**Keywords:** Empyema, Streptococcus pneumoniae serotype 9V, 13-valent pneumococcal conjugate vaccine

## Abstract

**Background:**

Clinical vaccine failure is the occurence of the specific vaccine-preventable disease in an appropriately and fully vaccinated person after enough time has elapsed for protection against the antigens of the vaccine to develop. Fully immunized cases with pneumoccal vaccine may sometimes develop a complicated pneumonia with empyema caused by a vaccine serotype.

**Case Report:**

A 2 year-old male patient was admitted with the complaints of fever. On the basis of findings and laboratory results, the patient was diagnosed as having empyema. He was successfully treated with parenteral antibiotics and chest tube drainage. The pleural fluid culture and hemoculture of the patient yielded penicillin-susceptible pneumococci and the isolate was identified as serotype 9V. The patient had been vaccinated with a 13-valent pneumococcal conjugate vaccine according to the Turkish national immunization schedule at 2, 4, 6 and 12 months of age. His medical history and basic immunological profile were inconsistent with a primary immunodeficiency.

**Conclusion:**

The failure of the PCV13 vaccine may results in a complicated pneumonia with empyema. It is important to investigate serotypes of pneumococci in these cases to determine other possible vaccine failures due to PCV13 and to study the underlying mechanisms.

Childhood pneumonia can be complicated by empyema leading to considerable morbidity and mortality. *Streptococcus pneumoniae* is still an important etiologic agent underlying empyema and other types of invasive pneumococcal diseases (IPDs) in children. Hence, 7-valent pneumococcal conjugate vaccine (PCV7) including serotypes 4, 6B, 9V, 14, 18C, 19F, and 23F was introduced in young children. However, IPDs due to other serotypes (e.g. 1, 7F and 19A), which are not involved in PCV7, also appeared and the need for a new vaccine with extended serotype coverage become evident. Therefore, 13-valent pneumococcal conjugate vaccine (PCV13), which includes additional serotypes (1, 3, 5, 6A, 7F and 19A) was developed. The Turkish national immunization schedule covered PCV7 at 2, 4, 6, and 12 months of age in 2008 and replaced it by PCV13 in 2011. An increase in empyema cases was observed after the implementation of PCV7 (1,2,3). Studies from Europe have reported empyema cases associated with both vaccine and non-vaccine serotypes (1). In a multicenter study from Turkey, which investigated the serotypes of *Streptococcus pneumoniae* that caused empyema in children between 2010 and 2012, the potential serotype coverages of PCV7 and PCV13 were found to be 16.3%, and 60%, respectively (4). None of the children were vaccinated in this study. On the other hand, a specific vaccine-preventable disease can occur in an appropriately and fully vaccinated person. This condition, which is defined as clinical vaccine failure, can be seen for various reasons. Here, we report such a patient who suffered from empyema due to *Streptococcus pneumoniae* type 9V in spite of completed immunization with PCV13.

## CASE PRESENTATION

**Figure 1:**
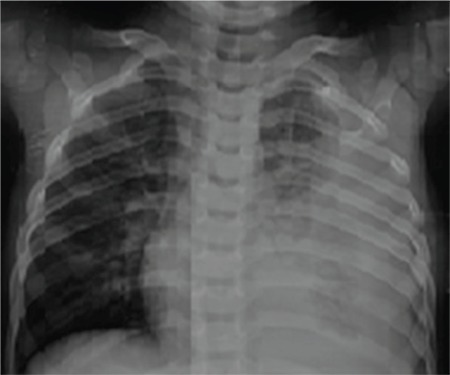
Pleural effusion and consolidation on chest X-ray of the patient.

**Figure 2:**
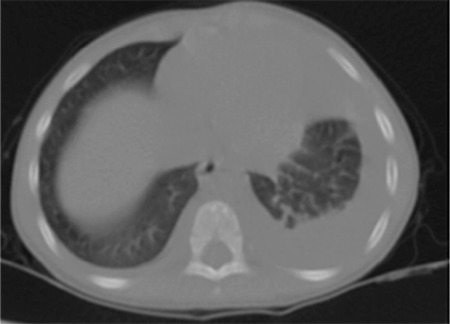
Pleural effusion from baseline to the left upper lobe and accompanying total collapse of the left lower lobe.

A 2 year-old boy was admitted to hospital with the complaints of fever and abdominal discomfort. His weight and height were 26 kg (50-75 p) and 122 cm (50-75 p), respectively. On physical examination, he was irritable, pale and febrile (temperature of 38.6 °C). The heart rate was 120/min and respiratory rate was 58/min with intercostal recession and diminished air entry on the left hemithorax on auscultation. Left-sided pleural effusion was suspected due to findings of the physical examination and it was confirmed on chest X-ray (Figure 1) and pleural ultrasonography (USG) which showed total collapse of the left lower lobe and pleural effusion from baseline to the upper lobe (Figure 2). A viscid purulent fluid was aspirated on pleural tap. Analysis of pleural fluid revealed an exudate with a total of 10200 cells/mm^3^ (84% polymorphs and 16% lymphocytes), protein 3 gr/dL, lactate dehydrogenase 1410 IU/L and glucose 17 mg/dL. An intercostal drain was placed and high dose intravenous ampicillin sulbactam (400 mg/kg/day) was initiated empirically. Total blood leukocyte count was 23.800/mm^3^ with a differential of 71% polymorphonuclear leucocytes, 18%, lymphocytes and 4% eosinophils. C-reactive protein was 261 mg/L (normal range 0-5 mg/L). Gram positive diplococci were seen on Gram stain of the pleural fluid. The pleural fluid culture and hemoculture of the patient yielded penicillin-susceptible *Streptococcus pneumoniae* and the isolate was identified as serotype 9V by the Quellung reaction using serotype-specific antisera (Statens Seruminstitut; Copenhagen, Denmark). Because the drainage stopped, the intercostal catheter was removed after 6 days. Ampicillin/sulbactam was replaced by teicoplanin after 9 days of treatment due to the incomplete resolution of pneumonic consolidation and the presence of minimal loculated fluid in the left pleural cavity seen on chest X-ray and pleural USG. On the 6th day of teicoplanin treatment, a marked regression of pathological findings occurred on pleural USG. No pathological findings were found on basic testing for primary immunodeficiency, which included measurement of immunoglobulin (Ig)G, IgA, IgM, and IgE levels and lymphocyte subset analysis. He was negative for anti-HIV. PCV13 vaccination of the patient was complete at 2, 4, 6, and 12 months of age. Total anti-pneumococcal IgG was detected as 2.2 microgr/mL (Binding site VaccZyme anti-PCP IgG EIA-kit; USA). The patient was discharged after 26 days of hospitalization. He is on follow-up for 6 months and doing well. Written informed consent has been obtained from the parent of the patient.

## DISCUSSION

*Streptococcus pneumoniae* is among the most common etiologic agents in invasive bacterial diseases of children like pneumonia, meningitis, and sepsis. Although empyema is a rare complication of pneumonia, it has become increasingly common for unclear reasons, as shown by several studies (2,3). Grijalva et al. (2) suggested that the increasing trend in empyema was also present before PCV7 introduction; for this reason, a direct association with the PCV7 vaccination program is unlikely. They also found an increase in the empyema cases due to other or unspecified pathogens among children aged <5 years (2). On the other hand, in a study from Utah, USA, it was found that the rate of pneumococcal parapneumonic effusion among all invasive pneumococcal diseases increased from 17.5% in the period before routine vaccination with PCV7 (1996 to 2000) to 32% after routine vaccination (3). The authors think that this observed increase in empyema cases is important and warrants continuous monitoring. Studies assessing the efficacy of PCV13 against pneumococcal parapneumonic effusion are limited. Moreover, efficacy may differ according to geographic locations due to variations in etiologic serotypes. Turkey has high vaccination rates. By the end of 2014, the vaccination rate of Turkish children with 3 doses of PCV13 was reported to be 96% (see: www.saglik.gov.tr/TR/dosya/1-99961/h/siy2014haberbulteni.pdf). Data regarding the prevalence and serotype distribution of pneumoccoccal parapneumonic effusions before the implementation of pneumococcal conjugate vaccine are limited in our country. Ceyhan et al. (4) conducted a multicenter study in Turkey to investigate the serotypes of Streptococcus pneumoniae that caused empyema in children between 2010 and 2011, 2 years after the implementation of PCV7 and before PCV13. *Streptococcus pneumoniae* was found to be the causative agent in 34% of 156 empyema cases. Serotypes could be specified in 62.3% of them and serotype 1 (14.5%), serotype 5 (12.7%) and serotype 3 (9.1%) were detected most commonly. Serotype 9V was present in only one case (4). It was reported to be responsible for pneumococcic parapneumonic effusions in the range from 3.7-15% in different time periods (3,5,6).

Vaccination failure can be due to vaccine failure or a failure to vaccinate, which means inappropriate administration of a vaccine for any reason. Vaccine failure can be host-related or vaccine-related. Host-related reasons may be a defined immunodeficiency or insufficient immune response like age-related maturation problems of immune responsiveness, waning immunity, suboptimal health status and immunological interference (7). The main vaccine-related reason is that vaccines are not 100% efficacious against all included antigens. Moreover, there may be incomplete coverage of strains, serotypes, genotypes, antigenic variants, or escape mutants, which can cause a vaccine preventable disease (7). The problems about manufacturing may also lead to vaccine-related vaccination failure. The definition of a confirmed clinical vaccine failure is the occurrence of the specific vaccine-preventable disease in an appropriately and fully vaccinated person after sufficient time has elapsed for protection against the antigens of the vaccine to develop (7). Based on this definition, our case is consistent with a confirmed clinical vaccine failure. Six months after the completion of a full 3+1 schedule with PCV13, he developed pneumococcal empyema due to serotype 9V, which is included in PCV13. We found the total anti-pneumococcal IgG of the patient in protective range. However, because we did not determine the serotype-specific antibody levels, we cannot define this case as an immunological vaccine failure, which is defined as a failure of the fully and appropriately vaccinated person to raise the accepted markers of protective immune responses (7). The basic immunological tests and medical history were not indicative of a primary immunodeficiency. However, the patient will be further investigated and followed-up cautiously regarding the possibility of a primary immunodeficiency.

Recently, the first report of a failure of PCV13 to prevent the development of complicated pneumonia caused by serotype 3 was published in Greece (8). The authors detected *Streptococcus pneumoniae* in 66% of parapneumonic effusions during 2012. The most frequently identified serotypes were serotype 3 (75%), serotype 19A (15%) and serotype 14 (5%). They reported that 5 children with empyema due to serotype 3 were fully immunized with PCV13. Another case of vaccination failure with PCV13 was reported shortly after this first report (9). They described a 3 year-old immunocompetent girl with complete immunization (2+1 schedule), who developed pneumococcal parapneumonic effusion due to serotype 3, 23 months after the booster injection (9). The authors of these two reports disputed the protection of PCV13 against serotype 3 based on their cases with clinical vaccine failure and the findings of PCV13 immunogenicity studies (10), which found lower pneumococcal anti-polysaccharide IgG antibodies developed against serotype 3 than other serotypes of the vaccine (8,9). Here, we reported another PCV13 vaccination failure for a different serotype, serotype 9V, in a fully immunized child who developed pneumococcic empyema. Although manufacturing-related problems and inappropriate administration for any reason cannot be totally excluded, this case may also indicate a problem in the effectiveness of the vaccine. Therefore, every effort should be made for the detection and monitoring of *Streptococcus pneumoniae* serotypes in complicated pneumonia and empyema cases and other invasive pneumococcal diseases, in order to gather information about vaccine failure related to PCV13 and studying the underlying mechanisms.
